# Implants talk to each-other: RF heating changes when two DBS leads are present simultaneously during MRI

**DOI:** 10.1109/EMBC40787.2023.10340769

**Published:** 2023-07

**Authors:** Bhumi Bhusal, Fuchang Jiang, Jasmine Vu, Pia Sanpitak, Laleh Golestanirad

**Affiliations:** Department of Radiology, Northwestern University, Chicago, IL 60611 USA.; Department of Biomedical Engineering, Northwestern University, Evanston, IL 60608 USA.; Department of Biomedical Engineering, Northwestern University, Evanston, IL 60608 USA.; Department of Radiology, Northwestern University, Chicago, IL, 60611, USA.; Department of Radiology and Department of Biomedical Engineering, Northwestern University, Chicago, IL 60611 USA

## Abstract

Deep brain stimulation (DBS) has proven to be an effective treatment for Parkinson’s disease and other brain disorders. The procedure often involves implanting two elongated leads aimed at specific brain nuclei in both the left and right hemispheres. However, evaluating the safety of MRI in patients with such implants has only been done on an individual lead basis, ignoring the possibility of crosstalk between the leads. This study evaluates the impact of crosstalk on power deposition at the lead tip through numerical simulation and phantom experiments. We used CT images to obtain patient-specific lead trajectories and compared the power deposition at the lead tip in cases with bilateral and unilateral DBS implants. Our results indicate that the RF power deposition at the lead tip can vary by up to 6-fold when two DBS leads are present together compared to when only one lead is present. Experimental measurements in a simplified case of two lead-only DBS systems confirmed the existence of crosstalk.

## I. Introduction

Deep brain stimulation (DBS) has emerged as an important therapy for patients with Parkinson’s disease and other neurological disorders [1–3]. DBS involves delivering electrical stimulation to specific subcortical regions in the brain through elongated leads, which are connected to an implantable pulse generator (IPG) placed in the clavicle region. Patients with DBS implants can highly benefit from magnetic resonance imaging (MRI)— a powerful imaging modality that provides an exceptional contrast when imaging soft tissues such as the brain. Indeed, MRI plays an ever-increasing role in the care of DBS patients, enabling verification of DBS targets, monitoring of treatment progress, and imaging for other non-DBS related indications [4–7].

The safety of patients with implanted conductive leads during MRI is a primary concern, with radiofrequency (RF) induced heating being the most significant issue [8–13]. The metallic leads interact with the transmit electromagnetic field, generating induced current along the leads that are dissipated into the tissue through the DBS electrodes, leading to increased specific absorption rate (SAR) at the lead-tissue interface. The heating at the lead tip is influenced by various factors such as transmit field distribution, lead’s internal geometry and material, dielectric properties of tissue surrounding the lead, and lead trajectory and dimension [8, 10, 12, 14–21]. However, most studies have focused on single leads, while realistic scenarios, such as with DBS patients, may involve two or more leads present simultaneously in the body. The RF heating at the lead tip results from resonating currents produced along the length of the lead, as the lead interacts with transmit RF fields acting as an antenna. The presence of neighboring leads can also affect this behavior, leading to differences in RF heating compared to when only one lead is present. Recent studies with different types of implants have reported significant changes in RF heating when two leads were present simultaneously compared to one lead at a time [22, 23]. This highlights the need for further examination of the impact of implant crosstalk on RF heating.

This study uses numerical simulations to evaluate the effect of RF coupling between two DBS lead models on the RF heating at the tip of each lead at RF frequencies of 64 and 123 MHz, representing 1.5 T and 3 T MRI respectively. Additionally, experimental measurements were performed at both 1.5 T and 3 T with simplified trajectories of lead-only system using two identical commercially available leads.

## II. Methods

### A. DBS Lead Models and Electromagnetic Simulations

**Figure 1:**
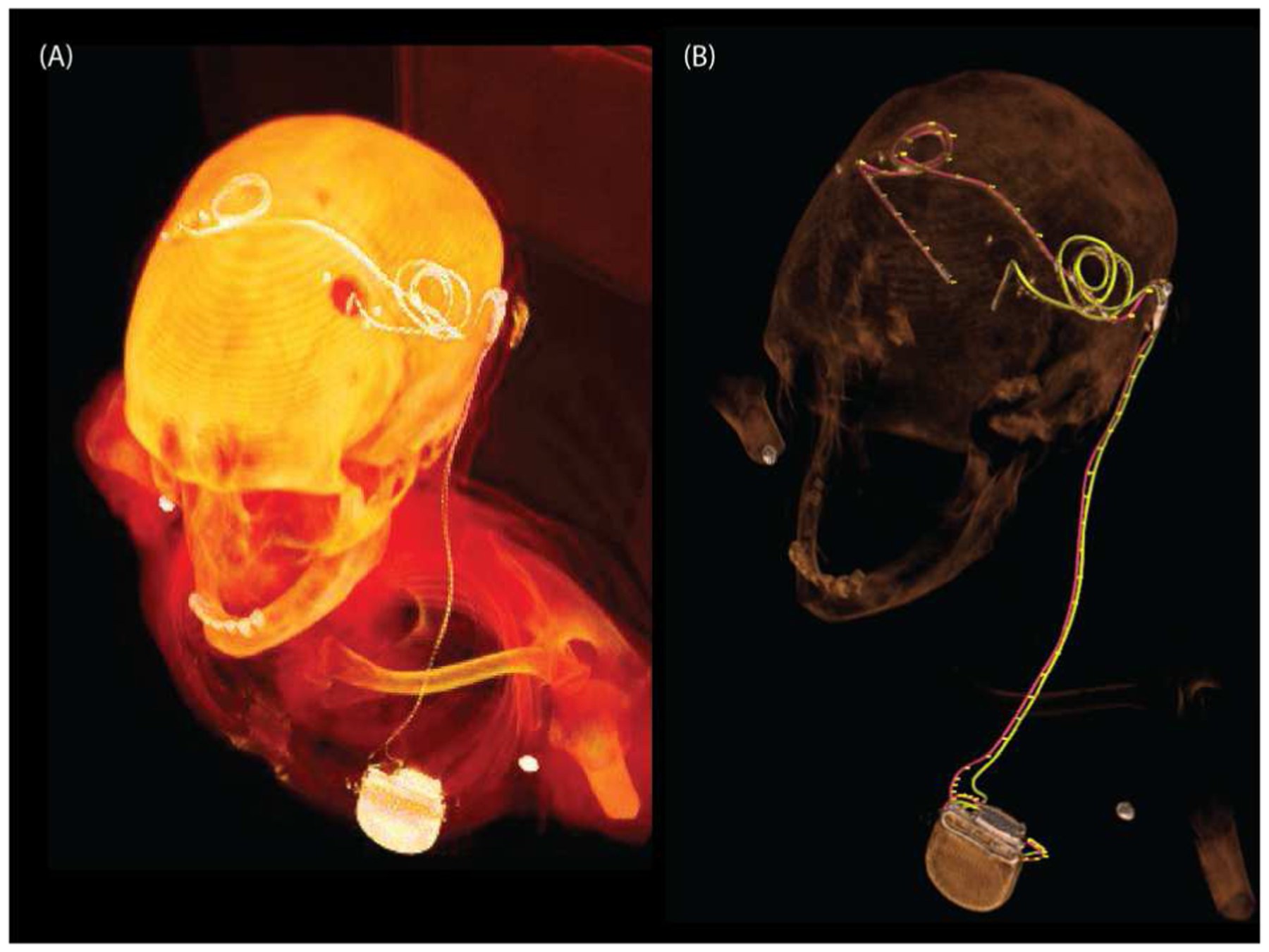
3D rendered view of CT image of a DBS patient with two DBS leads connected to single IPG (left) and segmented trajectories over the rendered view (right).

**Figure 2:**
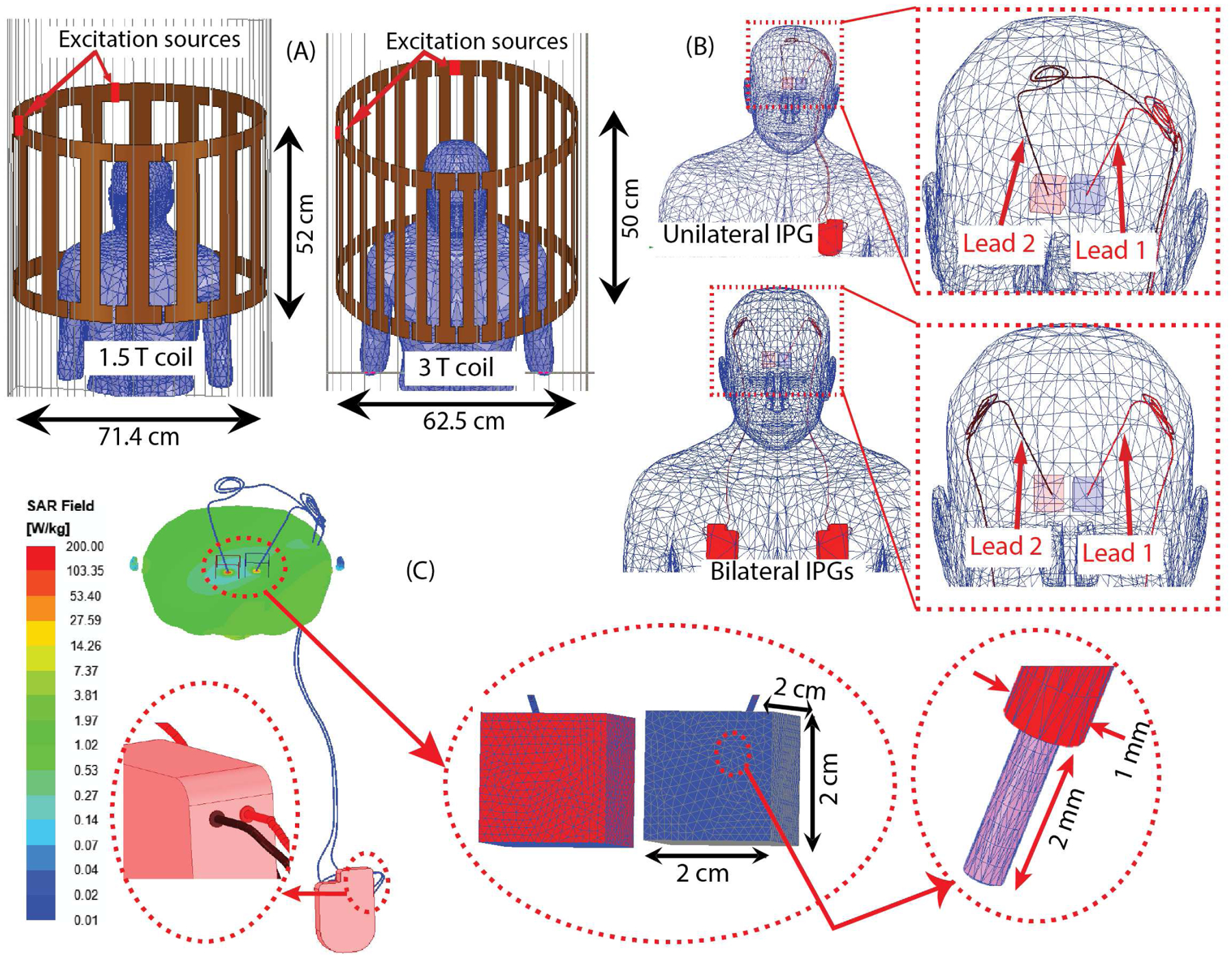
(A) Numerical simulation setup showing configurations of 1.5 T and 3 T coil. (B) Trajectories of Lead 1 and Lead 2 positioned inside the human body model showing unilateral as well as bilateral IPG cases. (C) Plot of 1gSAR on a transverse plane passing through lead tips for a unilateral case at 3 T, close view of mesh distribution in SAR boxes as well as lead tip and insulation and close view of lead-IPG interface. The conducting lead cores were electrically insulated from the IPG. The separation between the lead tips was ~2 cm for each case.

We developed patient-specific DBS lead trajectories based on CT images of a patient with two DBS leads implanted in the left and right subthalamic nucleus. The leads were connected to a double channel IPG placed in the left clavicle region (Fig. 1). We also created a scenario where each lead was connected to its own IPG by mirroring the IPG and the left lead (Fig. 2).

To evaluate RF heating, we used numerical simulations using HFSS (High Frequency Structure Simulator) module in Ansys Electronic Desktop 2021 R1 (Ansys Inc, PA, USA). We modeled the insulated wires (0.5 mm wire diameter, 0.25 mm insulation thickness, 2 mm exposed tip) based on the patient’s lead trajectories and placed them in a homogenous body model (conductivity σ = 0.47 S/m, relative permittivity ε_r_ = 80). The overall length of the wire was ~100 cm, typical of a DBS system with a 40 cm lead and a 60 cm extension.

We created models of birdcage transmit coils to represent a Siemens 1.5 T Aera body coil (16-rung, diameter =71.4 cm, length=52 cm) and a Siemens 3 T Prisma body coil (32-rung, diameter= 62.5 cm, length =50 cm). The coils were tuned to their respective frequencies (i.e., 64 MHz and 123 MHz) using a combined finite element method and circuit analysis as previously reported [24, 25]. The human body model was positioned in the coils with its head at the iso-center for all simulations.

We adjusted the input power of each coil to achieve a mean B_1_^+^ of 2 *μ*T on a circular axial plane (diameter = 5 cm) passing through the iso-center of the coil. We calculated the 1g-averaged SAR (referred to as 1gSAR) using the built-in SAR module in Ansys HFSS, following IEEE/IEC STD 62704–4 recommendations [26]. We recorded the maximum of 1gSAR (referred as Max1gSAR) in a cubic region (20 mm × 20 mm × 20 mm) surrounding the lead tip and compared the results of single vs double lead scenarios (Fig. 2). To improve simulation accuracy, we set the maximum mesh size to < 0.5 mm for the entire lead core, < 4 mm for the IPG, < 2 mm for the cubical tissue region surrounding the lead tip, and the lead insulation, and <20 mm for the body model. Simulations converged after 2–3 adaptive passes when the change in scattering parameters (ΔS) between two consecutive passes fell below a threshold of 0.02.

### B. Experiments

**Figure 3:**
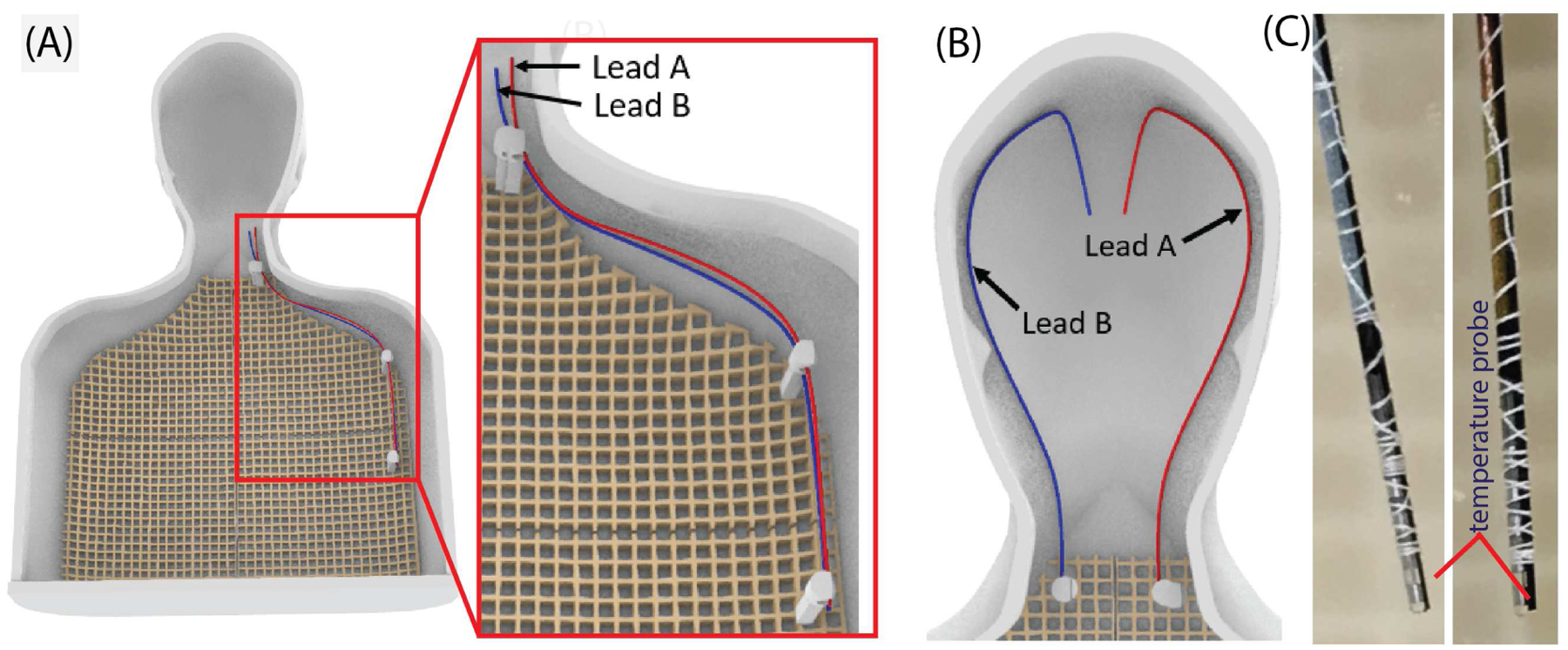
Experimental setup showing trajectories of leads (Lead A and Lead B) in human shaped phantom. (A) Trajectories of lead A and lead B placed together near left shoulder to maximize RF exposure. (B) Two leads placed bilaterally on left and right walls of phantom head and (C) Close view of each DBS lead tip connected to temperature probes. The phantom was filled with tissue mimicking gel and scanned at chest imaging landmark for setup (A) and head imaging landmark for setup (B). The separation between the lead tips was 1 cm for case (A) and 3 cm for case (B).

To determine if the crosstalk seen in the numerical simulations between wires could occur in real DBS leads, we conducted experimental measurements using two 40-cm commercial DBS leads (Lead model 6173, Abbott, TX, USA). The leads were implanted in a custom made human-shaped phantom filled with polyacrylic acid (PAA) gel (22 L). The gel was made by mixing 10 g/L polyacrylic acid salt powder (product no. 436364, Sigma Aldrich, Milwaukee, WI, USA) and 1.32 g/L NaCl in distilled water, resulting in a conductivity of σ= 0.47 S/m and a relative permittivity of ε_r_= 88 at 64 MHz, as measured by using a vector network analyzer (Keysight Technologies, Santa Rosa, CA) and a dielectric measurement kit (N1501A). MR compatible fiber optic temperature sensor probes (Osensa Inc, Burnaby, BC, Canada) were attached at the most distal contact of each of the DBS leads (Fig. 3C) to measure the temperature rise in the vicinity of the electrode.

To minimize uncertainties, we performed measurements for the simplified case of lead-only DBS systems, without extension cables and the IPG. The leads were either placed closely together along the left shoulder of the phantom, (Fig. 3A), or on the opposite lateral halves of the phantom’s head (Fig. 3B). The former represents a worst-case scenario, with leads exposed to maximum electric field [27] while the later represents a more realistic scenario. Both leads were capped at the proximal end where they would normally connect to the extension cable. The phantom was scanned with its chest or head at the iso-center depending on location of the leads (shoulder or head) in a Siemens 1.5 T Aera and a Siemens 3 T Prisma scanner. We used a high SAR T1-TSE sequence with TR = 897 ms, TE = 7.3 ms, Acquisition time = 280 s, Flip angle = 158° at 1.5 T and TR = 1450 ms, TE = 7.5 ms, Acquisition time = 451 s, Flip angle = 122° at 3 T. The sequences were adjusted to reach maximum allowed SAR at normal operating mode at head imaging landmark and the corresponding values of B_1_^+^rms were 4.3 μT at 1.5 T and 2.5 μT at 3 T.

Measurements were then repeated with one of the leads removed from the phantom setup to compare the RF heating of a single lead vs. double leads.

## III. Results

### A. Simulation Results: DBS Leads Connected to a Unilateral IPG

**Figure 4:**
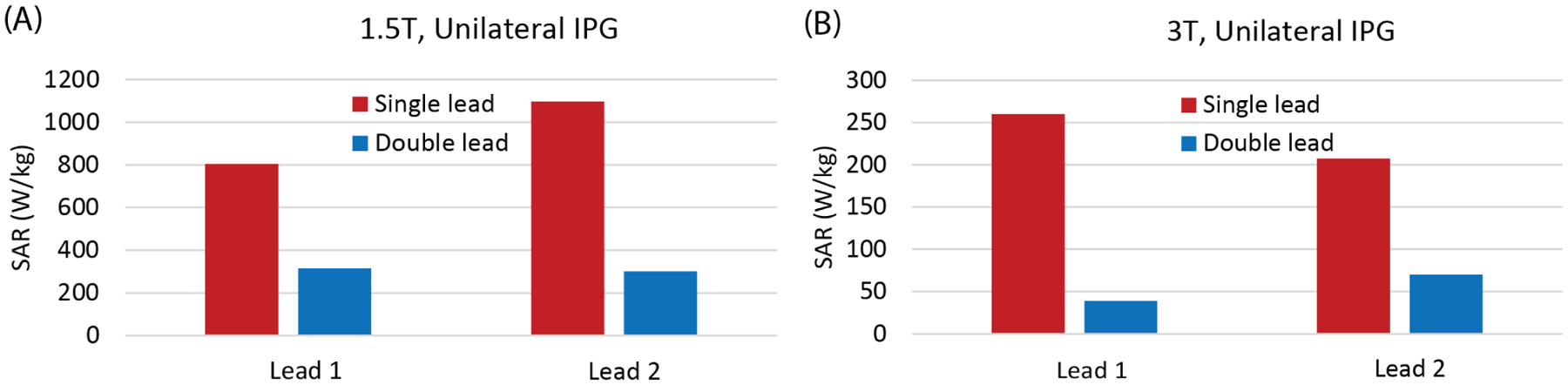
Max1gSAR at the tip of the two wire models connected to a single IPG placed in the left clavicle region. The input power of each coil was adjusted to achieve a mean B_1_^+^ = 2 μT on a central axial plane.

Fig. 4 presents the maximum of 1gSAR at the tips of wire models connected to a unilateral IPG exposed to RF fields at 64 MHz and 123 MHz. At 1.5 T, the Max1gSAR for Lead 1 decreased by more than 2.5-fold when Lead 2 was present. Similarly, the Max1gSAR around the tip of Lead 2 decreased by more than 3.6-fold when Lead 1 was present. The SAR variation showed a similar trend at 3 T, with a 6.7-fold decrease in Max1gSAR at the tip of Lead 1 and 3-fold decrease at the tip of Lead 2 when the leads were together compared to the SAR with one lead at a time.

### B. Simulation Results: DBS Leads Connected to Bilateral IPGs

**Figure 5:**
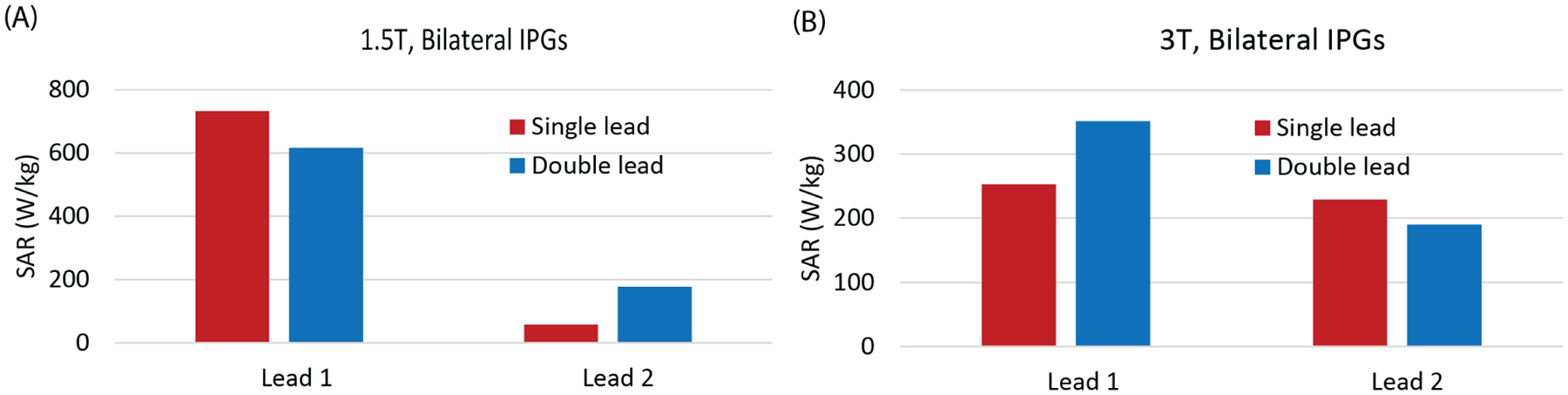
Max1gSAR at the tip of the two wire models each connected to an IPG placed in the clavicle region of respective side. The input power of each coil was adjusted to achieve a mean B_1_^+^ = 2 μT on a central axial plane.

Fig. 5 shows Max1gSAR at the tip of wire models connected to bilateral IPGs. At 1.5 T, the Max1gSAR for Lead 1 decreased by 16% when Lead 2 was present. Similarly, the Max1gSAR for Lead 2 increased by 3-fold when Lead 1 was present. At 3 T, the Max1gSAR at the tip of Lead 1 increased by 39% when Lead 2 was present, whereas the Max1gSAR at the tip of Lead 2 decreased by 17% when Lead 1 was present. The difference in RF heating for Leads on the left and right side can be understood from the left-right asymmetry in field distribution inside body due to deviation from cylindrical symmetry [27].

### C. Experimental Results

**Figure 6:**
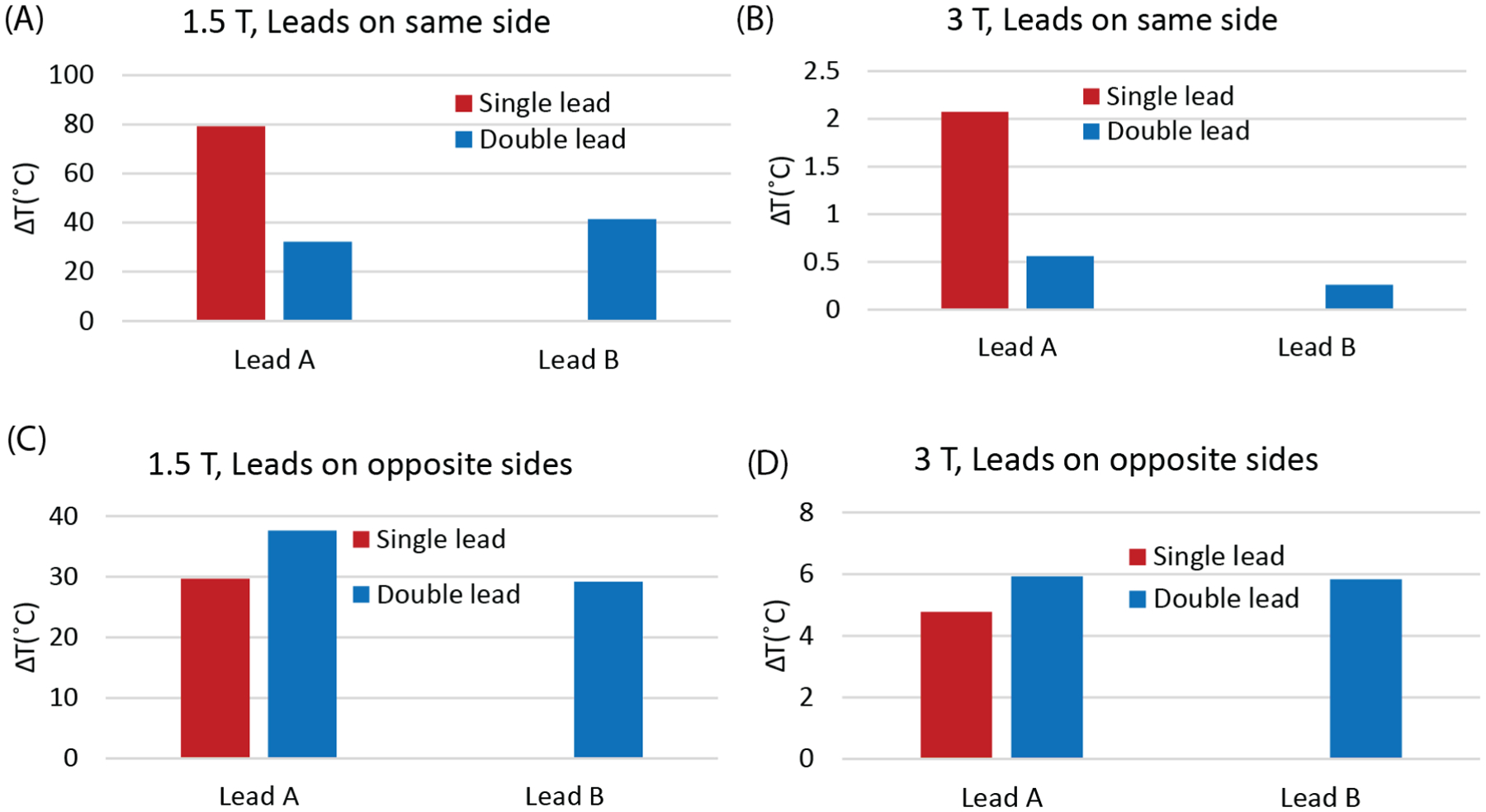
Temperature rise at the tip of DBS leads placed together in the left shoulder region (A & B) and placed on opposite side of head (C & D) during MRI scan at 1.5 T and 3 T. The leads placed in shoulder region were scanned at chest imaging landmark and the leads placed in head were scanned at head imaging landmark.

We observed a substantial crosstalk between commercial leads during MRI at 1.5 T and 3 T. Fig. 6 shows the temperature at the tip of leads for different configuration scenarios. When leads were on the same side (as shown in Fig. 3A), the temperature rise at the tip of Lead A increased by 2.5-fold and 3.7-fold at 1.5 T and 3 T MRI scans, respectively, when Lead B was removed. Conversely, when the leads were on opposite sides of the phantom (Fig. 3B), the temperature rise at the tip of Lead A decreased by 26% and by 24% at 1.5 T and 3 T scans, respectively, when Lead B was removed.

## IV. Discussion

Our findings suggest that the presence of two elongated leads can impact the RF heating during MRI. Both simulations and experiments demonstrate that the RF heating can be substantially altered when one of the two leads is removed, indicating significant crosstalk between the leads. The impact of crosstalk on the heating of each lead can either reduce or increase it, depending on the position and configuration of the leads. The increase or decrease in the RF heating of the leads can be explained by mutual impedance between the leads, which can increase or decrease the imput impedance of each lead acting as an antenna. This effect has been discussed in more detail in an earlier work [22].

When the leads are positioned next to each other, connected to a single IPG on the same side of the body, our results suggest that crosstalk may reduce the SAR. However, if the leads are positioned on opposite sides of the body and connected to IPGs on their respective sides, crosstalk may increase the SAR. It is important to note that the effects of crosstalk may depend on the lead trajectories and termination configuration as well as surrounding tissue properties, and further studies will be needed to generalize these findings. Our results are consistent with earlier study performed on different type of implants [22, 23].

## V. Conclusion

The crosstalk between the elongated implants like DBS can substantially alter the RF heating due to the implants during MRI scan. Possible worsening of the power deposition at the lead-tissue interface should be taken into consideration while evaluating safety of two or more implants during MRI.

## Clinical Relevance—

Our results indicate that RF heating of implanted leads during MRI can be affected by the presence of another lead in the body, which may increase or decrease the power deposition in the tissue depending on the position and configuration of the leads.
